# Confinement induces oxidative damage and synaptic dysfunction in mice

**DOI:** 10.3389/fphys.2022.999574

**Published:** 2022-11-24

**Authors:** Nuomin Li, Hao Wang, Shuchen Xin, Rui Min, Yongqian Zhang, Yulin Deng

**Affiliations:** ^1^ Institute of Engineering Medicine, Beijing Institute of Technology, Beijing, China; ^2^ School of Life Science, Beijing Institute of Technology, Beijing, China; ^3^ Beijing Key Laboratory for Separation and Analysis in Biomedicine and Pharmaceuticals, Beijing Institute of Technology, Beijing, China

**Keywords:** confinement, depressive-like behavior, prefrontal cortex, label-free quantitative proteomics, synaptic dysfunction

## Abstract

A confined environment is an enclosed area where entry or exit is highly restricted, which is a risk factor for a work crew’s mental health. Previous studies have shown that a crew is more susceptible to developing anxiety or depression in a confined environment. However, the underlying mechanism by which negative emotion is induced by confinement is not fully understood. Hence, in this study, mice were retained in a tube to simulate short-term confinement. The mice exhibited depressive-like behavior. Additionally, the levels of H_2_O_2_ and malondialdehyde in the prefrontal cortex were significantly increased in the confinement group. Furthermore, a label-free quantitative proteomic strategy was applied to analyze the abundance of proteins in the prefrontal cortex of mice. A total of 71 proteins were considered differentially abundant proteins among 3,023 identified proteins. Two differentially abundant proteins, superoxide dismutase [Mn] and syntaxin-1A, were also validated by a parallel reaction monitoring assay. Strikingly, the differentially abundant proteins were highly enriched in the respiratory chain, oxidative phosphorylation, and the synaptic vesicle cycle, which might lead to oxidative damage and synaptic dysfunction. The results of this study provide valuable information to better understand the mechanisms of depressive-like behavior induced by confined environments.

## 1 Introduction

A confined environment is a narrow, enclosed space, and these spaces often exist in manned aircraft, mines and vessels. In space, astronauts feel lonely in the confined environment of the spacecraft, which places serious psychological pressure on astronauts. The confined environment can cause astronauts negative emotions, such as tension, anxiety, and depression, which have an adverse effect on cognitive performance (Suedfeld and Weiss, 2000; Van Wijk, 2017). It is crucial to understand the effects of confined environments on the physiology and psychology of astronauts.

To better understand the effects of isolated and confined environments on negative emotions, the Mars-500 project simulated the complete journey from Earth to Mars in a confined 500 m^3^ cabin for 520 days in 2010 (Wang et al., 2014). The confined crew was susceptible to recording positive ratings for negative stimuli and exhibited a changing pattern of psychological adaptation over time during the 520 days of isolation. During prolonged confinement, a reduction in physical activity was associated with neuromuscular disorder (Belavy et al., 2013). Regarding the association of DNA methylation with phenotypes, three pathways enriched in glucose metabolism, mood state, and circadian rhythm were observed based on the Personalized Epigenetic-Phenotype Synchronization Analysis algorithm in the Mars-500 study (Liang et al., 2019). At the proteome level, seven proteins associated with inflammation were identified as indicators in 56 urine samples from Mars-500 volunteers (Khristenko et al., 2016). In addition, the rats exposed to simulated complex space environment were found that chronic stress induced depressive-like behaviors. The findings of membrane proteome implied that complex space environment interfered with the development of the nervous system, especially the regeneration of synaptic neurons (Cacioppo et al., 2009).

It was found that the incidence of mental illness among young people detained for violation of the law was much higher than that among their normal peers, which indicated that a confined environment may be detrimental to mental health and neurodevelopment (White et al., 2010; Wang et al., 2021). The brain structure and function of lonely young people tend to be impaired in confined environments (Cacioppo et al., 2009). Another study showed that submariners were susceptible to anxiety and depression in confinement (Van Wijk, 2017). As a kind of acute stress, short-term confinement induced the elevation of IL-6 levels in brown adipose tissue (Qing et al., 2020). It can also affect the decision-making ability of animals and promote the T-maze avoidance response (de Andrade et al., 2018). In the Lunar Palace 365-day experiment, natural light and perception were shown to have a positive effect on emotion in an isolated environment and to decrease the risk of anxiety and depression (Meng et al., 2020).

Despite the great advances that have been made, the neuromolecular mechanism of negative emotion during exposure to a confined environment remains largely unknown. It is generally acknowledged that the prefrontal cortex (PFC) plays a critical role in the regulation of emotion (Moda-Sava et al., 2019; Poleszak et al., 2019). Therefore, in this study, we used the quantitative proteomic strategy to explore the potential mechanism of confined space on oxidative stress and depression-like behavior in the brain. We hypothesized that confined environment would induce oxidative stress in the brain, and then induce the occurrence of depression-like behaviors by affecting synaptic function. The results of this study provide insights into the mechanism of depressive-like behavior induced by a confined environment and might offer useful clues for developing countermeasures to maintain mental health in a confined environment.

## 2 Materials and methods

### 2.1 Chemicals and reagents

Urea, dithiothreitol (DTT) and ammonium bicarbonate were purchased from Sigma‒Aldrich (Steinheim, Germany). The protease inhibitor was supplied by Roche (Mannheim, Germany). Sequencing-grade modified trypsin was purchased from Promega (Madison, WI). Mono Spin Centrifuge Columns were purchased from GL Sciences (C_18_, C_18_ FF, Ph, Tokyo, Japan). HPLC-grade formic acid and acetonitrile (ACN) were purchased from Fisher Scientific Canada (Edmonton, Canada). Water was obtained from a Milli-Q Plus purification system (Millipore, Bedford, MA, United States).

### 2.2 Animal model

C57BL/6J male mice (22 ± 1 g) were purchased from Beijing River Laboratory Animal Technology Co., Ltd. All experimental procedures were approved by the Ethics Committee of the Beijing Institute of Technology (Beijing, China) (Permit number: SYXK-BIT-20210118002), and the protocols were in accordance with the Beijing Institute of Technology Guide for the Care and Use of Laboratory Animals. Animals were housed individually in cages to adapt to laboratory conditions (12-h light-dark cycle and free access to water and food). The twenty mice were randomly divided into a control group (*n* = 9) and a confinement group (*n* = 11). The mice in the control group were maintained in normal cages and had free access to water and food. The confinement group was established by retaining each mouse in a ventilated 50 ml Falcon tube for 2 h (Domingues et al., 2019; Tu et al., 2019).

### 2.3 Behavioral studies

After 2 h of confinement, the mice were transferred to standard laboratory conditions. Behavioral tests, including tail suspension and forced swimming, were carried out to study the effects of confinement on the mice.

#### 2.3.1 Tail suspension test

The tail suspension test (TST) was used to assess the “desperate behavior” status of the mice. Mice were hung upside-down by a small metal hook and fixed with adhesive tape (2 cm from the tip of the tail) for 6 min. The immobility time during the last 4 min was recorded. Mice suspended by the tail attempted to escape but failed, and they eventually abandoned struggling and entered a depressed state (motionless and passive) (Can et al., 2012).

#### 2.3.2 Forced swimming test

The forced swimming test (FST) was used to assess the “desperate behavior” status of the mice. Each mouse was forced to swim in a cylindrical glass container (diameter, 20 cm; height, 50 cm) with 30 cm of water (22°C ± 1°C) for 6 min. The immobility time during the last 4 min was recorded. Immobility time was recorded when the hind limbs were not moving. The forepaws and tail still swayed slightly to maintain body balance (Porsolt et al., 1977).

### 2.4 Oxidative stress assay

After the behavioral tests, the mice were sacrificed by transcranial perfusion with precooled saline at 4°C. Then, the cortex tissues were dissected and preserved at −80°C for further research. The level of hydrogen peroxide (H_2_O_2_) in cortex tissues was determined using an H_2_O_2_ assay kit (Nanjing Jiancheng Bioengineering Research Institute, China) based on the molybdic acid colorimetric method. A malondialdehyde (MDA) assay kit (Nanjing Jiancheng Bioengineering Research Institute, China) was used to determine MDA levels. Data are expressed as the mean ± SD and were analyzed with GraphPad Prism 8 with an unpaired *t test*. A *p*-value less than 0.05 was considered statistically significant.

### 2.5 Proteomic analysis

#### 2.5.1 Sample preparation

Four samples from each group were randomly selected for proteomic research. The proteins in the prefrontal cortex (PFC) were extracted following the standard protocol with slight modification. The PFC was homogenized in ice-cold buffer [8 M urea, 10 mM DTT, 50 mM NH_4_HCO_3_ and a protease inhibitor mixture (Roche Diagnostics, Germany)], followed by ultrasonic disruption at a power of 28% (210 V) for 1 min (Uibra Cell, Sonics). The samples were centrifuged at 4°C and 12,000 × g for 30 min, and the supernatant was transferred to a new tube. The protein concentration was measured by the BCA method. Each sample was reduced for 30 min at 56°C, alkylated with 50 mM iodoacetamide in the dark for 30 min, and then diluted and digested for 16 h at 37°C by trypsin at an enzyme/protein ratio of 1:50. The residual trypsin activity was quenched by the addition of 3% formic acid (v/v). The peptides were desalted using a C_18_ solid phase extraction (SPE) column (C_18_, C_18_ FF, Ph, Tokyo, Japan) and dried using a vacuum centrifuge. The peptide concentration was determined by the BCA peptide assay.

#### 2.5.2 LC‒MS/MS analysis

The peptide mixture was dissolved in water containing 0.1% FA and analyzed using an online U3000-nano coupled with an Orbitrap Q-Exactive HF-X mass spectrometer (Thermo Fisher Scientific, Massachusetts, United States). Peptides were separated using a 15 cm house-made C_18_ reversed-phase column (100-μm inner diameter, 1.9 μm resin) and a 90 min elution gradient. Mobile phase A consisted of 0.1% FA and H_2_O, and mobile phase B consisted of 20% H_2_O and 80% ACN. A 90 min gradient (mobile phase B: 5.0% at 0 min, 10.0% at 16 min, 22.0% at 60 min, 35.0% at 78 min, 99.0% at 83 min, 99.0% at 85 min, 5.0% at 86 min, 0% at 90 min) was used at a flow rate of 300 nl/min. The data were acquired in data-dependent mode. For mass spectrometry parameters, the *m/z* range was set to 350–1,500 for the MS scan, and the accumulation time was 0.25 s. The top 20 most intense ions in MS1 were selected for MS/MS analysis, and the dynamic exclusion time was 20 s.

#### 2.5.3 Maxquant data processing

The RAW mass spectrometry files were processed using MaxQuant with an integrated Andromeda search engine using a false discovery rate (FDR) < 0.01 at the protein and peptide levels. Tandem mass spectra were searched against the UniProt Mouse database (15/12/2019, taxonomy ID: 10090, 55474 sequences) concatenated with a reverse decoy database. The following parameters were used: 20 ppm first search peptide tolerance, 4.5 ppm main search peptide tolerance; trypsin enzyme specificity, a maximum of two missed cleavages; fixed modification: carbamidomethyl (C), variable modification: oxidation (M); and the option of match between runs was enabled with a matching time window of 0.7 min and an alignment window of 20 min. The other parameters in MaxQuant were set to the default values. The built-in label-free quantification algorithm (LFQ) in MaxQuant was applied for quantification.

### 2.6 Data analysis and visualization

The MaxQuant search results were imported into Perseus (v.1.6.2.3) software for statistical analysis. First, the rows marked as “Reverse,” “Potential contaminants,” and “Proteins only identified by site” were excluded. The rows with no missing values across all six samples were conserved for subsequent analysis to achieve high stringency, and the LFQ intensity values were log-transformed with base 2. Then, the DAPs were identified by unpaired two-tailed Student’s *t test* in Perseus using cutoffs set to an FDR of 0.05 and an S_0_ value of 1. DAVID 6.8 bioinformatics tools (https://david.ncifcrf.gov/) were used to assign functional annotations to the differentially abundant proteins (DAPs). Gene ontology (GO) biological process (GOBP), GO cellular components (GOCC) and GO molecular function (GOMF) terms were identified with FDR < 0.05. Moreover, we used Cytoscape (version 3.6.1), the plugin ClueGO (version 2.5.4) and Cluepedia (version 1.5.4) to display the Kyoto Encyclopedia of Genes and Genomes (KEGG) pathways and protein‒protein interactions (PPIs) of related proteins. A two-sided hypergeometric test with the Benjamini‒Hochberg correction was performed to assess enrichment significance. Only pathways with *p* values < 0.05 are presented. The kappa score was set to 0.7, which was calculated based on the number of proteins shared between pathways, as an indicator of grouped pathways. The PPI information was generated from the STRING database. To reduce data redundancy, the kappa score of the PPI was also set to 0.7.

### 2.7 Parallel reaction monitoring analysis

The DAPs between the confinement and control groups were selected for targeted quantification by parallel reaction monitoring (PRM) analysis. Skyline software (MacCross Laboratory, University of Washington) was utilized to analyze the peak area generated from precursor ions in both the control and confinement groups (MacLean et al., 2010). Relative protein quantification was calculated by the ratio of the peptide peak area of the confinement group to that of the control group.

### 2.8 Western bolt analysis

The PFC protein were solubilized in RIPA lysis buffer with protease inhibitors. The protein concentrations were analyzed through Pierce BCA protein quantitation assay kit (Thermo Fisher Scientific, Massachusetts, United States). The proteins were separated with 12% SDS-PAGE gel and then transferred by electroblotting onto a 0.22 μm polyvinylidene fluoride (PVDF) membrane (Bio-Rad, United States). After blocking in 5% skim milk for 2 h at room temperature, the membrane was incubated with primary antibody (anti-syntaxin1A; anti-synaptophysin, anti-Tubulin, proteintech, United States) at 4°C overnight. Furthermore, the blot was incubated with HRP-conjugated goat Anti-Rabbit IgG (H + L) or HRP-conjugated goat Anti-Mouse IgG (H + L) (ZSGB-BIO, China) for 2 h at room temperature. Blots were developed using an ECL Kit (Millipore, United States) following the manufacturer’s protocol.

## 3 Results

### 3.1 Behavioral tests

**FIGURE 1:**
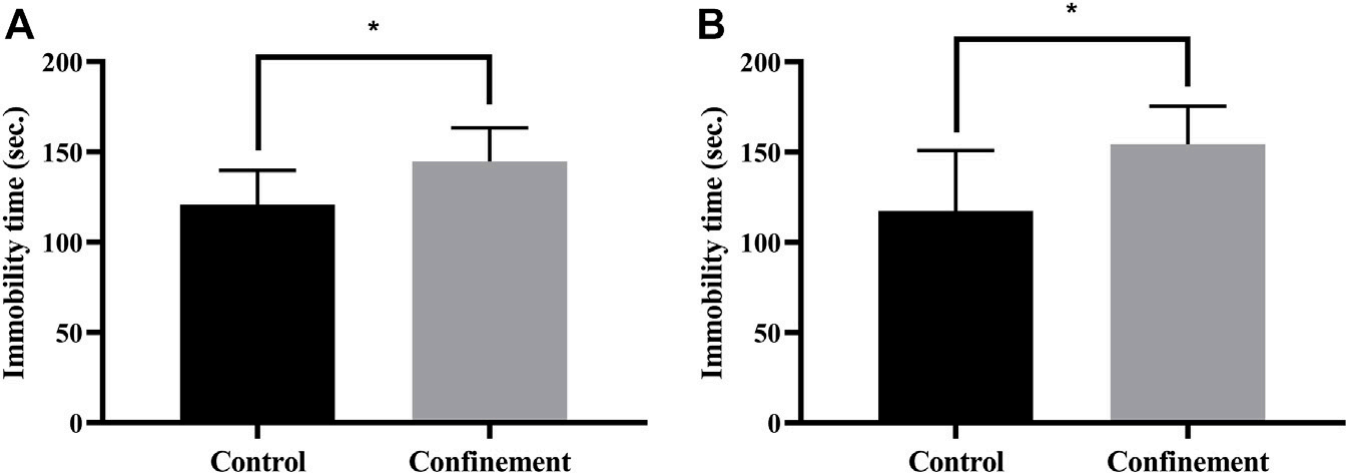
Effect of confinement on the behaviors of rats. **(A)** Tail suspension test (control: *n* = 9, confinement: *n* = 11). **(B)** Forced swimming test (control: *n* = 9, confinement: *n* = 11). Data are expressed as the mean ± SD, and the *p*-value was determined by an unpaired Student’s *t* test. *p < 0.05, vs. control group.

To investigate the effect of confinement on mice, the tail suspension test (TST) and forced swimming test (FST) were performed. As shown in Figure 1A, the immobility time of the confinement group was significantly increased compared with that of the control group in the TST. Furthermore, in the FST, the immobility time was also significantly elevated in the confinement group (Figure 1B). These results demonstrated that the mice exhibited depressive-like behaviors induced by confinement.

### 3.2 Oxidative stress

**FIGURE 2:**
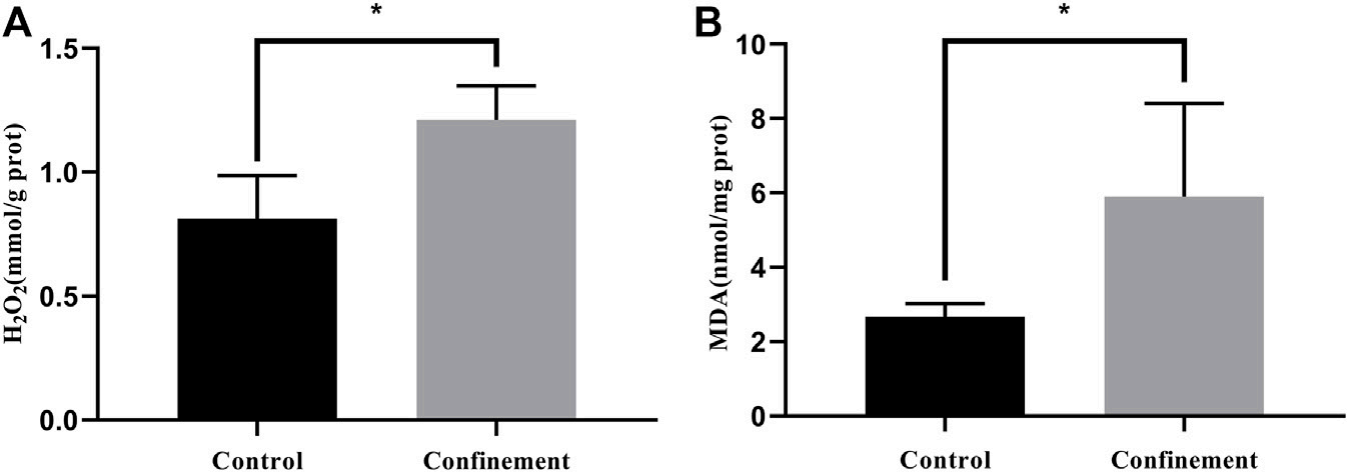
The level of oxidative stress was evaluated in both the control and confinement groups. **(A)** The level of H_2_O_2_ was significantly increased in the confinement group. **(B)** The concentration of MDA was dramatically elevated in the confinement group. Data are presented as the mean ± SD in mice (*n* = 8). Statistical significance was measured using an unpaired Student’s *t* test. *p < 0.05, vs. control group.

The prefrontal cortex (PFC) is an important cortical region that integrates information from numerous cortical and subcortical regions. It plays critical roles in cognitive functions, emotional regulation and sociability. Dysfunction of the PFC has been found in various neurological and psychiatric disorders, such as schizophrenia, depression, Alzheimer’s disease and addiction (Xu et al., 2019). To further investigate whether confinement induces oxidative stress in the PFC, H_2_O_2_ and MDA were determined in both the control and confinement groups. As shown in Figure 2A, the H_2_O_2_ concentration in the cerebral cortex of the confinement group was significantly increased compared with that in the control group. However, the level of MDA increased in the confinement group (Figure 2B). Therefore, the results indicated that confinement stress might disrupt the redox balance of the cerebral cortex.

### 3.3 Comparative proteomic analysis of the prefrontal cortex during confinement

**FIGURE 3:**
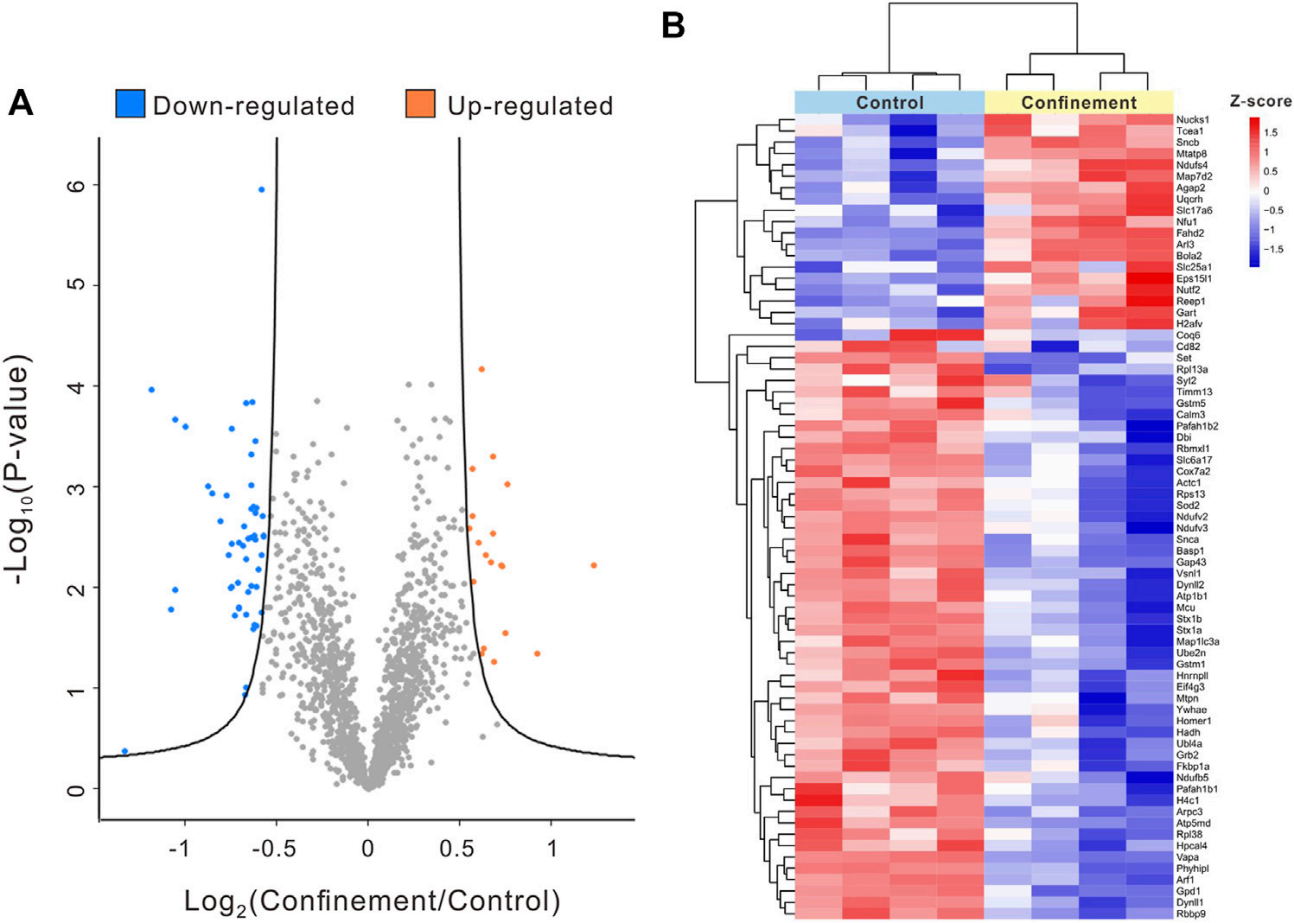
Comparative proteomic analysis of the PFC of mice in the control and confinement groups. **(A)** Volcano plot depicting a total of 3,023 proteins identified and quantified in the proteomic analysis of the control and confinement groups. The significant DAPs in blue dots and orange dots represent downregulated and upregulated proteins in the confinement group, respectively (S_0_ = 1; FDR = 5%). The non-significant proteins are shown in gray. **(B)** Heatmap of DAPs: The red and blue blocks represent 19 upregulated proteins and 52 downregulated proteins in the confinement group, respectively. The color of the blocks represents the fold change.

A total of 3,023 proteins were identified using a quantitative proteomic strategy (Supplementary Table S1). To obtain the significant DAPs, a volcano plot was plotted using cutoffs set with an FDR of 0.05 and an S_0_ value of 1. As shown in Figure 3A, 71 proteins were quantified as DAPs between the control and confinement groups. Among these DAPs, 19 proteins were upregulated, and 52 proteins were downregulated in the confinement group (Supplementary Table S2). A heatmap was generated to show the expression levels of DAPs between the control group and the confinement group (Figure 3B). The functional annotations of the DAPs were studied to probe clustering information, such as “biological process,” “cellular component,” and “molecular function.”

**FIGURE 4:**
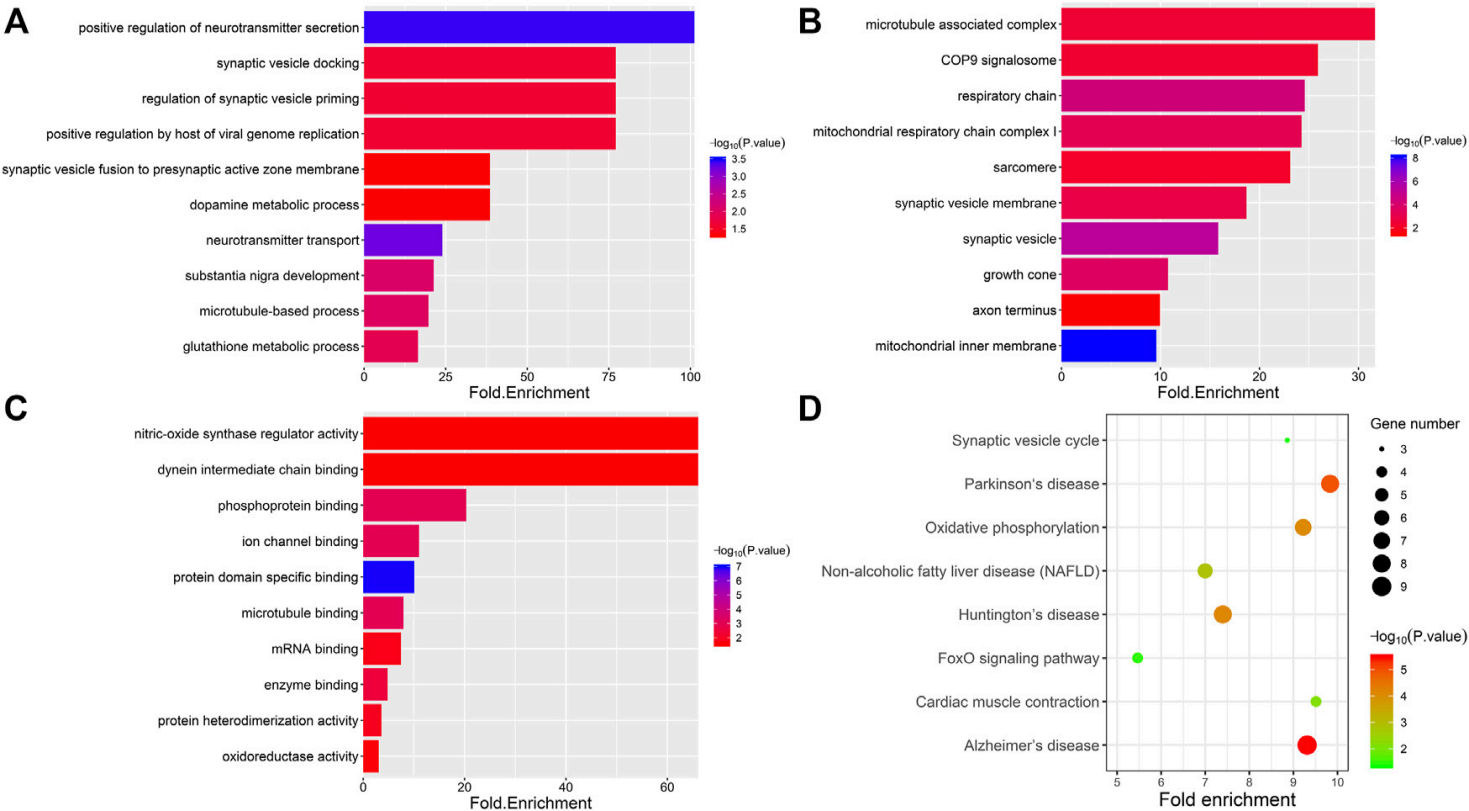
Gene ontology and KEGG pathway enrichment analysis of differentially expressed proteins. Four categories were evaluated: biological process (BP), cell composition (CC), molecular function (MF) and KEGG pathway. The top 10 significantly enriched GO annotations associated with DAPs belonging to the categories biological process **(A)**, cell composition **(B)**, and molecular function **(C)**. **(D)** The top eight enriched pathways from KEGG analysis are shown along with the *p*-value and gene number. The color represents the significance of enrichment.

To obtain functional information about the DAPs, GO and KEGG analyses were performed using the DAVID bioinformatics tool with a *p*-value less than 0.05; the analysis included four categories: biological processes, cellular components, molecular functions and KEGG pathways (Figures 4A–D). The top five terms in biological processes were positive regulation of neurotransmitter secretion, synaptic vesicle docking, regulation of synaptic vesicle priming, positive regulation by host of viral genome replication and synaptic vesicle fusion to presynaptic active zone membrane. Regarding cellular components, the DAPs were enriched in microtubule-associated complex, COP9 signalosome, respiratory chain, mitochondrial respiratory chain complex I and sarcomere. In the molecular function category, DAPs were highly enriched in nitric-oxide synthase regulator activity, dynein intermediate chain binding, phosphoprotein binding, ion channel binding and protein domain specific binding. To further analyze the key enriched pathways of the DAPs, KEGG analysis was performed, as shown in Figure 4D. It was observed that the DAPs were involved in the synaptic vesicle cycle, Parkinson’s disease, oxidative phosphorylation, nonalcoholic fatty liver disease (NAFLD) and Huntington’s disease. As shown in Table 1, 10 proteins in total were associated with oxidative stress and synaptic dysfunction.

**TABLE 1:** The DAPs involved in oxidative stress and synaptic dysfunction. Fold Change = Confinement/Control.

Accession	Protein name	Difference (Log_2_ fold change)	Protein score	Sequence coverage (%)	Peptide number
Q9CXZ1	NADH dehydrogenase [ubiquinone] iron-sulfur protein 4, mitochondrial	0.60	24.09	29.7	9
P99028	Cytochrome b-c1 complex subunit 6, mitochondrial	0.56	323.31	58.4	9
O55042	Alpha-synuclein	−0.57	323.31	72.9	17
Q9D6J6	NADH dehydrogenase [ubiquinone] flavoprotein 2, mitochondrial	−0.61	158.88	57.7	12
Q9CQH3	NADH dehydrogenase [ubiquinone] 1 beta subcomplex subunit 5, mitochondrial	−0.63	89.10	26.5	5
P09671	Superoxide dismutase [Mn], mitochondrial	−0.64	127.54	57.2	12
O35526	Syntaxin-1A	−0.68	208.20	51.4	15
Q8BK30	NADH dehydrogenase [ubiquinone] flavoprotein 3, mitochondrial	−0.73	42.02	36.5	2
P48771	Cytochrome c oxidase subunit 7A2, mitochondrial	−0.74	24.08	27.7	3
P61264	Syntaxin-1B	−0.77	323.31	55.6	23

### 3.4 Protein‒protein interaction and biological pathway networks

**FIGURE 5:**
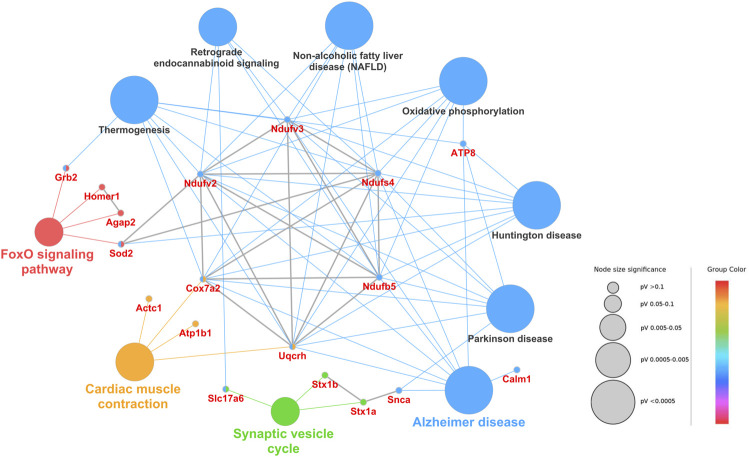
Protein‒protein interaction and biological pathway networks of DAPs from the control and confinement groups. The node size represents the percent of associated genes of proteins per node, and the different pathways are displayed in different colors. Furthermore, the protein‒protein interaction networks were constructed using the Cluepedia plugin and STRING database with a STRING score threshold of 0.7. The protein‒protein interactions are marked as gray lines.

The protein interactome and various cellular pathways play indispensable roles in cell signaling, metabolism, regulation and homeostasis in live cells. Therefore, protein‒protein interaction and biological pathway networks were constructed for the DAPs to better understand how the proteins work together in confinement. As shown in Figure 5, the DAPs between the confinement group and the control group were enriched in ten pathways, including the FoxO signaling pathway, oxidative phosphorylation, Parkinson’s disease, Alzheimer’s disease, and the synaptic vesicle cycle.

### 3.5 Validation of DAPs by parallel reaction monitoring

Label-free quantitative proteomic analysis allowed us to identify and quantify 71 DAPs between the control and confinement groups. To validate the accuracy of the label-free method, the downregulated proteins superoxide dismutase [Mn] (Sod2) and syntaxin-1A (Stx1a) were arbitrarily selected and quantified by PRM analysis. As listed in Table 2, the fold changes of the two proteins obtained by PRM analysis was consistent with the label-free results. To further validate the results from both in parallel reaction monitoring and label-free quantification using mass spectrometry, we performed the Western blot to quantify one significantly down-regulated protein syntaxin-1A and one unchanged protein synaptophysin in Supplementary Figure S1. It indicated that the findings between the mass spectrometry and Western blot were basically consistent.

**TABLE 2:** Comparison of fold changes of proteins both in parallel reaction monitoring and label-free quantification.

Protein name	Selected peptide sequence	Precursor	Product	y/b ion series	PRM fold change(Log_2_ Confinement/Control)	Label-free fold change(Log_2_ Confinement/Control)
		(*m/z*)	(*m/z*)			
Superoxide dismutase [Mn], mitochondrial (Sodm)	GDVTTQVALQPALK	720.90, z^2+^	839.53+	y8	−0.64	−0.62
			740.46+	y7		
			428.28+	y4		
	AIWNVINWENVTER	872.44, z^2+^	1,160.56+	y9		
			1,047.48+	y8		
		581.96, z^3+^	747.36+	y6		
			618.32+	y5		
Syntaxin-1A (Stx1a)	SIEQSIEQEEGLNR	816.39, z^2+^	1,174.56+	y10	−0.55	−0.66
			974.45+	y8		
			845.41+	y7		
		544.60, z^3+^	588.31+	y5		
			459.26+	y4		
	QALSEIETR	523.77, z^2+^	734.36+	y6		
			518.29+	y4		
			451.26+	y3		

## 4 Discussion

The environment experienced by astronauts in space consists of multiple factors, including confinement, microgravity, radiation and noise (Garrett-Bakelman et al., 2019). Previous studies have demonstrated that isolation and confinement adversely impact a crew’s mental health and performance, which may indirectly affect mission success. However, the neural mechanism induced by confinement remains largely unknown. In this study, we retained mice in tubes for 2 h to establish an acute stress model. The mice exhibited depressive-like behavior in a confined space. Furthermore, oxidative stress tests showed that the H_2_O_2_ and MDA levels were increased, which was deleterious for neurons. Then, a label-free quantitative proteomic strategy was applied to identify and quantify the DAPs between the control and confinement groups. Of 3,023 identified proteins, a total of 71 proteins were considered DAPs. Bioinformatic analysis revealed that these DAPs were related to oxidative phosphorylation, the synaptic vesicle cycle, etc. The results suggested that oxidative damage and synaptic dysfunction contributed to confinement-induced depressive-like behaviors in mice. However, this short duration prevents elucidation of the underlying mechanisms in response to long-term confinement. Understanding of the effects of chronic confinement is also important for understanding changes in astronauts’ moods and performance during prolonged manned space flight.

### 4.1 Oxidative damage to synapses

As shown in Figure 5, the DAPs were enriched in the oxidative phosphorylation pathway. Among these proteins, Ndufv2, Ndufv3, Ndufb5, and Cox7a2 were downregulated, and Uqcrh6 was downregulated in the confinement group. As the key components of mitochondrial complex I, these proteins play essential roles in the respiratory chain. They can transfer an electron from NADH to CoQ, pump four protons from the mitochondrial matrix to the membrane space, and drive the synthesis of ATP (Formosa et al., 2018). The loss of mitochondrial complex I might decrease ATP generation, cause mitochondrial dysfunction and affect the proper function of synapses. Homer protein homolog 1 (Homer1) is a postsynaptic density scaffolding protein and may be involved in synaptic plasticity and development (Bridi et al., 2020). The downregulation of Homer1 indicated that the integrity and function of synapses might be disrupted. Grb2 and Sod2 were downregulated in confinement and were enriched in the FoxO signaling pathway. The FoxO signaling pathway is involved in many cellular physiological events, such as oxidative stress resistance, glucose metabolism and apoptosis (Mouchiroud et al., 2013). It was reported that the suppression of Grb2 expression could increase oxidative stress in cells (Shan et al., 2013). Superoxide dismutase [Mn](Sod2) in the mitochronia was an antioxidant protein that played fundamental roles in superoxide disproportionation and the rapid conversion of O^2^− to H_2_O_2_ (Xiu et al., 2020). Therefore, we proposed that the downregulation of Grb2 and Sod2 might contribute to the elevation of oxidative stress. Hydrogen peroxide and malondialdehyde (MDA) are known as important markers for oxidative stress (Liu et al., 2015). In our study, the data showed that the levels of H_2_O_2_ and MDA were both increased in the confinement group, as reported by others (From the American Association of Neurological Surgeons AANS et al., 2018; Sacks et al., 2018; Kanis et al., 2019). Excessive hydrogen peroxide has strong oxidation activity and produces high levels of radicals in the cell, which results in oxidative damage to proteins and membrane lipids. The downregulation of superoxide dismutase [Mn] leads to the elevation of radical levels within the cell. Excessive radicals can attack membrane polyunsaturated lipids and disrupt the structure and integrity of the cell membrane. Consequently, MDA, one of the final products of radical-induced lipid peroxidation, was also observed to increase in the confinement group. Previous studies also demonstrated that damage to synapses was associated with the elevation of H_2_O_2_ and MDA levels (Wang et al., 2016; Zhu et al., 2018). Our data suggested that confinement-induced oxidative stress might trigger neuronal damage, which likely plays a role in eliciting depressive-like behaviors in mice (Sacks et al., 2018; Casaril et al., 2021).

### 4.2 Synaptic dysfunction in mice

Previous studies revealed that synaptic dysfunction contributed to pathological development of depression (Duman and Aghajanian, 2012). In the confinement group, some DAPs were enriched in the synaptic vesicle cycle pathway. Syntaxin1a and Syntaxin1b are vital components of the SNARE complex, which participates in mediating the fusion of synaptic vesicles and presynaptic membranes (Costa et al., 2019; Vardar et al., 2020). The synaptic vesicle cycle was an essential step for synapse transmission between neurons (Melland et al., 2021). Syntaxin-1 was important for neuronal maintenance and survival. Reduction of Syntaxin-1A resulted in cessation of action potential evoked, spontaneous and a 50% reduction in morphologically docked vesicles (Vardar et al., 2016). Syntaxin-1A gene knockout mice exhibited abnormal behavior in fear conditioning and social and object recognition. In our study, the levels of Syntaxin-1A were significantly downregulated in the confinement group, suggesting that confinement reduced the stability of vesicles, inhibited the synaptic vesicle cycle. Consequently, synaptic transmission and plasticity were impaired in the confinement group. Additionally, another downregulated protein, α-synuclein, in the confinement group attracted our attention, since it is a hallmark of Parkinson’s, Alzheimer’s disease and potentially in inflammation-induced depression (Dantzer et al., 2021). α-Synuclein is highly expressed in presynaptic terminals and is involved in the process of synaptic vesicle recycling (Cheng et al., 2011). It was demonstrated that α-synuclein regulated synaptic plasticity by inducing vesicle aggregation. Furthermore, α-synuclein was directly implicated in neurotransmitter release and recycling as a molecular chaperone for SNARE complex assembly. The loss of α-synuclein might prevent synaptic vesicle recycling and cause synaptic dysfunction.

## 5 Conclusion

A confined environment is a risk factor for mental health issues in manned aircraft. A crew is susceptible to developing anxiety or depression in a confined space. In this study, mice were retained in a tube to simulate short-term confinement stress. Consequently, the mice exhibited depressive-like behaviors. The levels of H_2_O_2_ and malondialdehyde in the prefrontal cortex were significantly increased in the confinement group. Moreover, a label-free quantitative proteomic strategy was used to analyze the abundance of proteins in the prefrontal cortex of the mice. Of 3,023 identified proteins, 12 proteins were upregulated and 59 proteins were downregulated in the confinement group. Two differentially expressed proteins, superoxide dismutase [Mn] and syntaxin-1A, were also validated by a parallel reaction monitoring assay. Strikingly, the DAPs were highly enriched in oxidative phosphorylation, Alzheimer’s disease, Huntington’s disease, synaptic vesicle cycle, etc. The results indicated that confinement-induced oxidative stress and synaptic dysfunction contributed to depressive-like behaviors in mice.

## Data Availability

The datasets presented in this study can be found in online repositories. The names of the repository/repositories and accession number(s) can be found in the article/Supplementary Material.

## Supplementary material

SUPPLEMENTARY FIGURE S1 Analysis of the relative abundance of Styntaxin1A and Synaptophysin **(A)** Western blot results showed that the content of Styntaxin1A and Synaptophysin **(B)** Quantification analysis of protein Styntaxin1A and Synaptophysin 5(*n* = 3 per group). Data were expressed as mean ± SD and *p*-value was determined by an unpaired Student’s *t* test. **p < 0.05*, vs. control group.

SUPPLEMENTARY TABLE S1 All identified proteins in the left medial prefrontal cortex of mice.

SUPPLEMENTARY TABLE S2 Differentially abundant proteins in the comparison confinement vs. control Declarations.
