# Supplementary figures and images for: Confinement induces oxidative damage and synaptic dysfunction in mice
Source: Front Physiol. 2022 Nov 24;13:999574. doi: 10.3389/fphys.2022.999574 (PMC9729776; doi:10.3389/fphys.2022.999574)

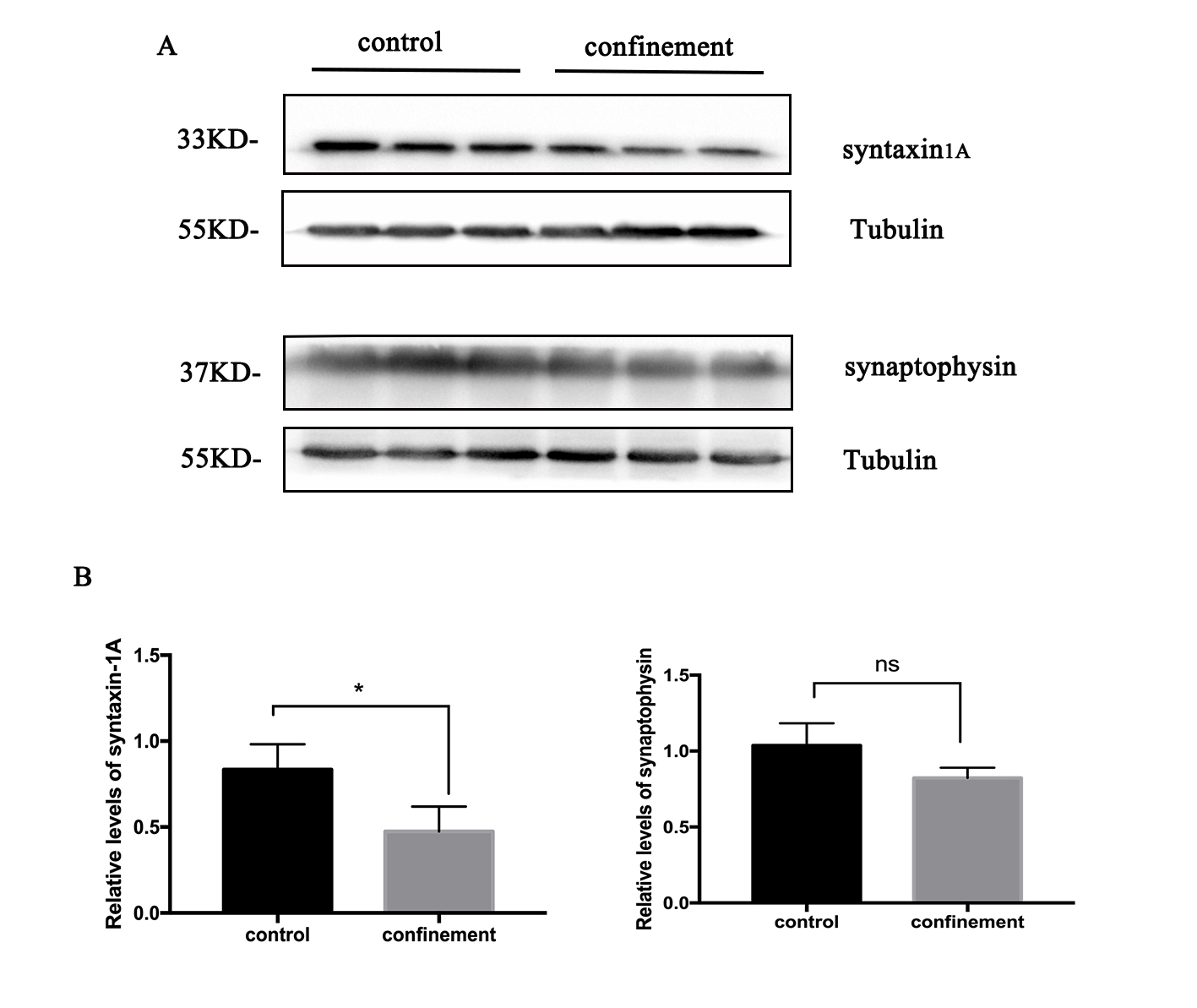

Supplement: Supplementary file 3 [file Image1.JPEG]
